# Immunomodulatory effects of alpha vs beta radiopharmaceutical therapy in murine prostate cancer

**DOI:** 10.3389/fimmu.2025.1563387

**Published:** 2025-05-22

**Authors:** Carolina A. Ferreira, Hemanth K. Potluri, Mojdeh Mahmoudian, Christopher F. Massey, Joseph J. Grudzinski, Amanda M. Carston, Nathan B. Clemons, Malick Bio Idrissou, Anna S. Thickens, Zachary T. Rosenkrans, Cynthia Choi, Caroline P. Kerr, Anatoly N. Pinchuk, Ohyun Kwon, Justin J. Jeffery, Bryan P. Bednarz, Zachary S. Morris, Jamey P. Weichert, Douglas G. McNeel, Reinier Hernandez

**Affiliations:** ^1^ Departments of Radiology, Pharmacology and Toxicology and Biomedical Engineering, Michigan State University, East Lansing, MI, United States; ^2^ Department of Medical Physics, University of Wisconsin-Madison, Madison, WI, United States; ^3^ Department of Medicine, University of Wisconsin-Madison, Madison, WI, United States; ^4^ Department of Cell and Molecular Biology, Michigan State University, East Lansing, MI, United States; ^5^ Departments of Medical Physics and Radiology, University of Wisconsin-Madison, Madison, WI, United States; ^6^ Pharmaceutical Sciences Program, University of Wisconsin-Madison, Madison, WI, United States; ^7^ Department of Human Oncology, University of Wisconsin-Madison, Madison, WI, United States; ^8^ Carbone Cancer Center, University of Wisconsin-Madison, Madison, WI, United States

**Keywords:** immunomodulation, tumor microenvironment, targeted alpha therapy, prostate cancer, radiopharmaceutical therapy, radionuclides

## Abstract

**Background:**

Radiation therapy can modulate the tumor microenvironment (TME), influencing antitumor immune responses. This study compared the immunomodulatory effects of alpha-emitting (^225^Ac) and beta-emitting (^177^Lu) radiopharmaceutical therapies (RPT) using NM600 in murine prostate cancer models.

**Methods:**

We assessed immunological changes in TRAMP-C1 and Myc-CaP tumor models treated with ^225^Ac-NM600 or ^177^Lu-NM600. Flow cytometry was used to profile immune cell populations, activation markers, and checkpoint molecules, while multiplex assays analyzed cytokine and chemokine expression.

**Results:**

In general, ^225^Ac-NM600 elicited stronger immunomodulatory effects than ^177^Lu-NM600, including cell line dependent increased CD8/Treg ratios, activation of effector and memory T cells, and depletion of suppressive Tregs and MDSCs. The treatment elevated Th1 cytokines, pro-inflammatory chemokines, and checkpoint molecules like PD-1 on CD8+ T cells and PD-L1 on MDSCs, creating a more “hot” TME.

**Conclusion:**

Alpha-emitting ^225^Ac-NM600 demonstrated superior ability to enhance antitumor immunity compared to beta-emitting ^177^Lu-NM600. These findings support the use of ^225^Ac-NM600 in combination with immunotherapies for advanced prostate cancer treatment.

## Graphical Abstract

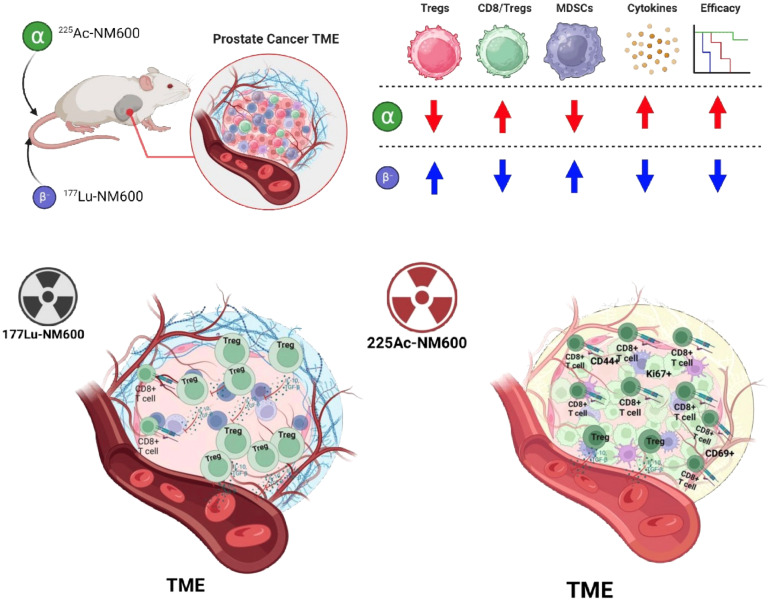

## 1 Introduction

Prostate cancer (PCa) represents a critical global health issue, claiming over 350,000 lives annually. Due to its unique reliance on androgens for growth and progression, androgen deprivation constitutes the foundational approach to PCa management. Its growth and progression are driven by androgens, positioning androgen deprivation therapy (ADT) as the primary treatment strategy. While effective initially, ADT often loses efficacy as most patients eventually develop metastatic castration-resistant prostate cancer (mCRPC), a condition with a mortality rate exceeding 50% and a median survival of less than three years (1). Despite advancements in therapeutic approaches, including the development of PARP inhibitors and radiopharmaceutical therapies (RPT) targeting the prostate-specific membrane antigen (PSMA), mCRPC remains an incurable disease (2–4). The ineffectiveness of current therapies in mCRPC can be partly attributed to the immunosuppressive tumor microenvironment (TME) characteristic of advanced PCa. This TME hinders immune responses and diminishes the effectiveness of immunotherapies. Radiation therapy, a staple of cancer treatment, has shown potential in modulating the TME to enhance antitumor immune responses (5, 6). However, external beam radiotherapy is limited in its application due to toxicities associated with treating large radiation fields, which curtail its ability to address metastatic disease effectively (7). Conversely, RPT enables the systemic delivery of radioactive agents that selectively induce cytotoxic DNA damage in tumor cells, making it particularly suitable for metastatic conditions (7). The clinical potential of RPT was highlighted by the FDA approval of ^177^Lu-PSMA-617 for PSMA-positive mCRPC patients, which demonstrated significant biochemical and radiographic responses (8). However, the therapy provides a modest survival benefit, extending life by only four months on average. Moreover, treatment outcomes are inconsistent, with 10-20% of patients showing limited responses due to moderate or heterogeneous PSMA expression, and approximately one-third of patients failing to respond altogether despite adequate PSMA levels (9, 10). High-grade hematologic toxicity remains another concern, with severe cytopenias reported in up to 9% of patients, particularly those with diffuse red marrow infiltration (11).

To improve outcomes, there is growing interest in combining novel treatments or developing agents that target alternative biomarkers. Among emerging approaches, targeted alpha therapy (TAT) has attracted considerable attention due to the favorable dosimetric properties of alpha-emitting radionuclides. Compared to beta emitters, alpha particles deliver higher linear energy transfer (LET), shorter particle range, and greater relative biological effectiveness (RBE), offering the potential for improved therapeutic efficacy (12). Promising preclinical and clinical data have been reported for PSMA ligands radiolabeled with alpha emitters such as ^225^Ac, ^212^Pb, and ^213^Bi (12). Despite these advances, systematic studies examining the differential radiobiological effects of alpha and beta emitters in RPT for PCa are scarce (13, 14). Research focusing on these effects within the context of a functional immune system is even more limited.

Our group has developed NM600, an alkylphosphocholine (APC) analog, which demonstrates high tumor uptake and retention across multiple cancer models, including PCa (15, 16). We have exploited the capacity of cancer cells to selectively sequester and retain metabolically resistant phospholipids (15) to develop NM600, an APC showing elevated tumor uptake and retention in multiple tumor models, including PCa (16). When labeled with beta emitters such as ^90^Y and ^177^Lu, NM600 significantly inhibited tumor growth and prolonged survival in preclinical cancer models (6, 17). Notably, ^90^Y-NM600 showed immunomodulatory effects that synergized with immune checkpoint inhibition (ICI) therapy in immunologically “cold”—defined as poorly responsive to immunotherapy—melanoma, breast cancer, neuroblastomas, and head and neck cancer (18, 19). However, in a recent study (20), we demonstrated that ^90^Y-NM600 treatment led to the enrichment of immunosuppressive Tregs in the TME that negated efficacy in combination with anti-PD1 ICI in murine models of PCa. Given the experience with External Beam Radiotherapy (EBRT), radiation’s biological and immunomodulatory effects depend on several factors, including dose, fractionation, and the type of radiation used (21, 22). To address these factors, we investigated the antitumor and immunomodulatory properties of ^177^Lu-NM600 and ^225^Ac-NM600 in syngeneic TRAMP-C1 and Myc-CaP murine PCa models. The TRAMP-C1 and Myc-CaP murine prostate cancer models were carefully selected to reflect clinically relevant subtypes of advanced prostate cancer, ensuring that findings from this study can be meaningfully translated into patient care. The TRAMP-C1 model, derived from the Transgenic Adenocarcinoma of the Mouse Prostate (TRAMP) model, represents an androgen-sensitive tumor that undergoes progression toward castration resistance, mimicking the natural disease course observed in patients with metastatic castration-resistant prostate cancer (mCRPC). This transition from an androgen-dependent state to therapy resistance makes TRAMP-C1 particularly useful for investigating radiopharmaceutical therapy (RPT) in the mCRPC setting, where standard treatments, such as ADT and second-generation antiandrogens, eventually fail. By contrast, the Myc-CaP model, which originates from a Myc-driven adenocarcinoma, retains androgen receptor (AR) expression and signaling, making it an ideal model for studying AR-driven prostate cancers, which remain a major therapeutic challenge due to their heterogeneous responses to RPT and immunotherapy. By employing these two immunocompetent murine models, this study allows for a detailed comparison of the effects of ^225^Ac-NM600 and ^177^Lu-NM600 in distinct biological contexts, including differences in tumor immunogenicity, radiation sensitivity, and immune response dynamics. These models not only enable a mechanistic evaluation of the immunomodulatory effects of alpha- and beta-emitting radionuclides but also provide insights into how TME factors, such as immunosuppressive cell infiltration and cytokine expression, influence treatment outcomes. Understanding these interactions is crucial for optimizing RPT regimens, particularly in combination with ICIs or other targeted therapies aimed at overcoming resistance mechanisms (21, 22). Key to this investigation was estimating the tumor dosimetry for both isotopes to facilitate a systematic comparison of their effects. Our findings demonstrated that ^225^Ac-NM600 offers several advantages over its beta-emitting counterpart. Specifically, the alpha emitter exhibited superior antitumor efficacy due to its higher LET and RBE, increased cytotoxicity, and enhanced ability to stimulate pro-inflammatory cytokine expression. Herein, we uncovered ^225^Ac-NM600‘s significant advantages in antitumor efficacy stemming from marked cytotoxicity and ability to stimulate pro-inflammatory cytokine expression and reduce immunosuppressive cell lineages (i.e., Tregs and MDSCs) within the TME. Additionally, ^225^Ac-NM600 significantly reduced immunosuppressive cell populations, including Tregs and myeloid-derived suppressor cells (MDSCs), within the TME. The findings of our research provide compelling evidence for the therapeutic potential of ^225^Ac-NM600, particularly in combination with immunotherapies, for the treatment of advanced PCa.

## 2 Materials and methods

### Animal studies

Two distinct murine prostate cancer cell lines were utilized in this study: TRAMP-C1, derived from transgenic C57BL/6 mice, and Myc-CaP, originating from FVB mice. Both cell lines were acquired from ATCC, cultivated in DMEM supplemented with specific nutrients, including glucose, L-glutamine, sodium bicarbonate, and sodium pyruvate. The cells were maintained in an environment with 5% CO_2_ at 37°C. Animal experiments were conducted in accordance with the guidelines set by the Institutional Animal Care and Use Committee (IACUC) at the University of Wisconsin. Male C57BL/6 and FVB/NJ mice, aged 6 weeks, were obtained from Jackson Laboratories and allowed to acclimate for one week. For tumor induction, TRAMP-C1 cells were injected subcutaneously into the right flank of C57BL/6 mice, while Myc-CaP cells were similarly inoculated into FVB/NJ mice. The cell suspension for injection consisted of 1 x 10^6 cells in 100μL of sterile PBS, mixed in a 1:1 ratio with Matrigel. Tumors were permitted to grow to approximately 200 mm^3 before the mice were used in biodstribution or therapy studies. Tumor growth was monitored by measuring tumor volumes three times per week using a digital caliper. The volume was calculated using the ellipsoid volume formula (5).

### Radiochemistry

To radiolabel 2-(trimethylammonio)ethyl(18-(4-(2-(4,7,10-tris(carboxymethyl)-1,4,7,10-tetraazacyclododecan-1-yl)acetamido)phenyl)octadecyl) phosphate (NM600), either ^177^Lu or ^225^Ac was mixed with the NM600 compound (10-100 µg per mCi per mCi of 177Lu or uCi of 225Ac). This reaction occurred in a 0.1 M sodium acetate buffer (NaOAc) with a pH of 5.5 and was heated at 90°C for 30 minutes. After labeling, the compounds were purified through reverse-phase chromatography using a Waters Oasis HLB Light system. Elution was performed with 100% ethanol, followed by drying under a stream of nitrogen (N_2_). The dried product was then reconstituted in a solution composed of 0.9% sodium chloride (NaCl) and 0.4% v/v Tween 20. Determination of yield and purity was accomplished using instant thin-layer chromatography (iTLC) with 50 mM EDTA and silica-impregnated paper (Perkin Elmer). A cyclone phosphor image reader analyzed the resulting chromatograms, showing that the labeled compound stayed at the origin (spotting point), while free radiometals traveled with the solvent front. Stability of ^225^Ac-NM600 was studied in 100% human serum for up to 168h. Samples were maintained shaking at 37°C. At different time points, aliquots were taken and iTLC was performed and analyzed.

### *Ex vivo* biodistribution

Mice bearing Myc-CaP (FVB/NJ) or TRAMP-C1 (C57BL/6) subcutaneous tumors were injected with ^177^Lu-NM600 (0.27 µg/MBq) or ^225^Ac-NM600 (0.027 µg/MBq). Culling occurred at 4, 24, 72, 120, and 192 hours post-injection (n=3 per group), and organs were collected for biodistribution analysis. For ^225^Ac-NM600, tissues were stored overnight to achieve secular equilibrium with ^213^Bi. Organs were weighed, analyzed via a gamma counter, and decay-corrected to calculate the percent injected activity per gram (%IA/g) %IA/g for each tissue.

### Dosimetry estimations

*Ex vivo* biodistribution data (**Supplementary Figure S1**) were used to estimate ^225^Ac-NM600 dosimetry. Organ masses were allometrically scaled for each mouse model, and residence times were determined via trapezoidal integration, assuming physical decay post-final time point. Dose factors were calculated assuming organs as self-dosing spheres. Total absorbed doses accounted for contributions from the complete decay chain of ^225^Ac, excluding redistribution of daughter isotopes. No adjustments for relative biological effectiveness (RBE = 1) were applied (**Supplementary Tables S1**, **S2** **).**

### Tumor microenvironment immunomodulation

Flow cytometry was performed on tumors collected from TRAMP-C1 or Myc-CaP tumor-bearing mice treated with ^177^Lu-NM600 (5.55 or 18.5 MBq) or ^225^Ac-NM600 (7.4 or 18.5 KBq) at days 7, 14, and 28 (n=3 per group). Tumors were processed, digested, and stained for immune markers (e.g., CD11b, CD4, CD8, CD25, Foxp3), and analyzed using fluorescence controls on an Attune NxT cytometer and FlowJo v10. Cytokine and chemokine analysis was conducted using ProcartaPlex panels, with tumor lysates prepared in NP40 detergent. Samples were analyzed on a Luminex MAGPIX system, and all tumors were below the 2500 mm³ humane endpoint for tumor volume.

### Statistical analysis

Quantitative data were analyzed using GraphPad Prism, with results expressed as mean ± standard deviation. Two-way ANOVA or unpaired t-tests were used for group comparisons, with statistical significance set at p < 0.05.

## 3 Results

### 3.1 Radiochemistry, biodistribution analysis and dosimetry estimations

Radiochemistry and stability of ^177^Lu-NM600 have been extensively studied previously (6). ^225^Ac-NM600 radiolabeling yield was consistently above 95%. ^225^Ac-NM600 maintained at high percentage of bound activity (around 90-95%) when incubated in human serum for up to 168 hours (7 days), suggesting good stability under physiological conditions (**Supplementary Figure S7**). Dosimetry analysis based on *ex vivo* biodistribution data revealed distinct absorbed dose patterns for ^177^Lu-NM600 and ^225^Ac-NM600 across TRAMP-C1 and Myc-CaP tumor models. **Supplementary Table S1** shows that for ^177^Lu-NM600, tumor absorbed doses were higher than most normal tissues, with values of 5.3 Gy (low dose) and 17.982 Gy (high dose) in TRAMP-C1 tumors, and 3.289 Gy (low dose) and 11.063 Gy (high dose) in Myc-CaP tumors. Tumor-to-normal tissue dosimetry ratios were favorable, particularly when compared to organs like the liver, spleen, and kidneys, which received higher doses due to hepatobiliary clearance. **Supplementary Table S2** highlights the superior but comparable dosimetric profile of ^225^Ac-NM600. Tumor absorbed doses were significantly higher relative to normal tissues, with values of 4.277 Gy (low dose) and 10.926 Gy (high dose) in TRAMP-C1 tumors, and 1.823 Gy (low dose) and 4.559 Gy (high dose) in Myc-CaP tumors. Notably, the liver received the highest absorbed doses among normal tissues due to prolonged retention of the radiopharmaceutical, reaching up to 24.6199 Gy in TRAMP-C1 models at high doses. **Supplementary Figure S1** illustrates the biodistribution patterns of both radiopharmaceuticals over time in TRAMP-C1 and Myc-CaP tumor-bearing mice. For ^177^Lu-NM600 (**Supplementary Figure S1A**), tumor uptake peaked at 24 hours post-injection in both models, with sustained retention observed up to 72 hours. Tissue uptake was predominantly observed in the liver, lungs, kidneys, and spleen due to hepatobiliary clearance mechanisms. For ^225^Ac-NM600 (**Supplementary Figure S1B**), tumor uptake was similarly high. The liver showed consistently high uptake across all time points, reflecting its role in clearance pathways. Temporal biodistribution differences between the two radiopharmaceuticals were found. For ^177^Lu-NM600, liver uptake peaked early at 24 hours post-injection and gradually declined by 72 hours. In contrast, ^225^Ac-NM600 exhibited sustained liver accumulation over extended periods, with peak uptake observed between 120 and 192 hours. This disparity is likely attributable to the unique decay properties of ^225^Ac, which releases multiple alpha-emitting daughter radionuclides during its decay chain. These daughters, such as ^213^Bi, may escape from the chelator due to recoil energy and accumulate in organs like the liver, which serves as a primary site for clearance and metabolism.

#### 3.1.1 ^225^Ac-NM600 Increases CD8/Treg Ratios and Activates Cytotoxic T Lymphocytes (CTLs) in Myc-CaP and TRAMP-C1 Tumors

**Figure 1:**
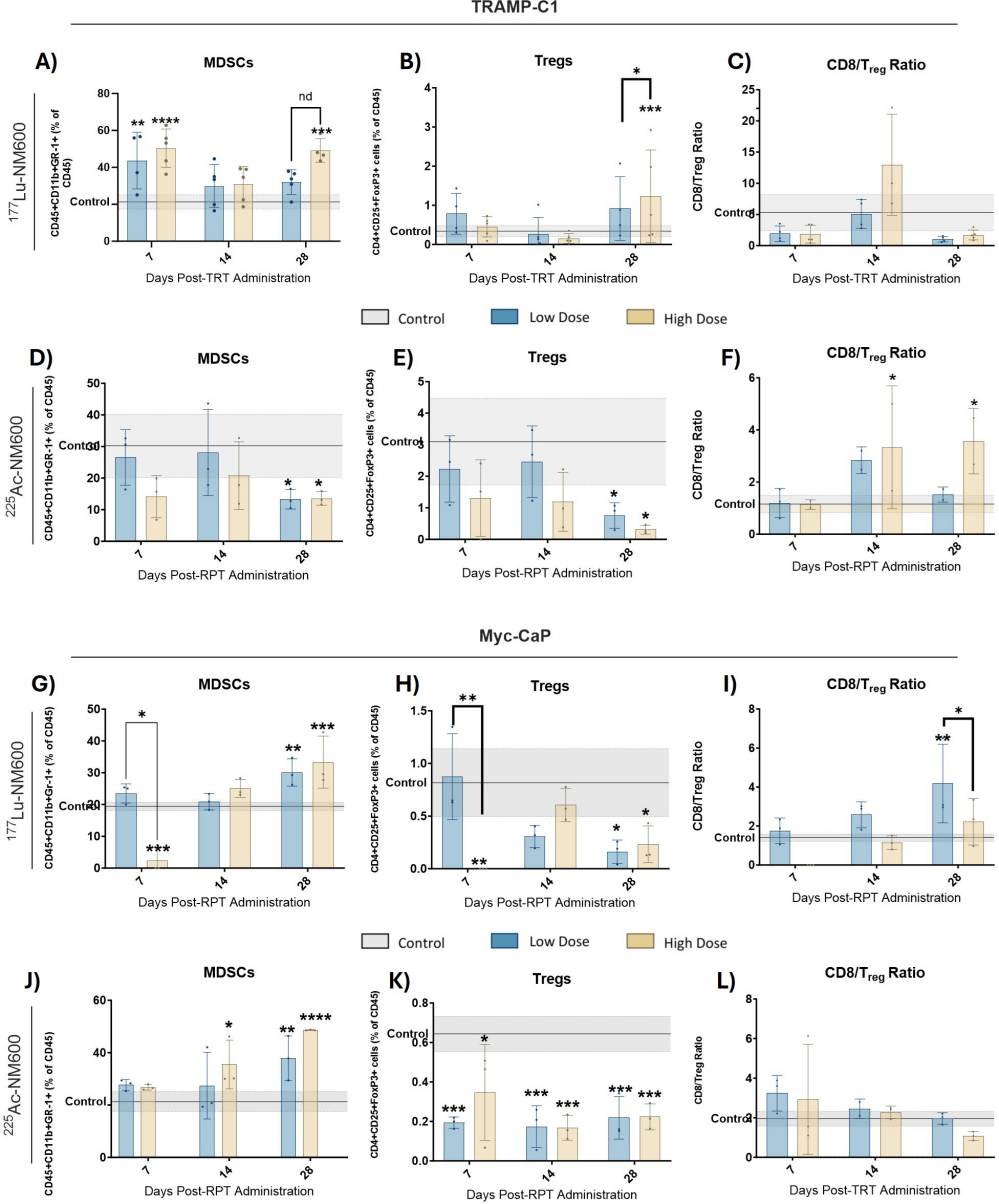
Flow Cytometry analysis of MDSCs and Treg markers in TRAMP-C1 tumor bearing mice receiving ^177^Lu-NM600 **(A-C)** or ^225^Ac-NM600 **(D-F)** and in Myc-CaP tumor bearing mice that received ^177^Lu-NM600 **(G-I)** or ^225^Ac-NM600 **(J-L)**. Statistical analysis compares to controls or otherwise noted. *p<0.05, ** p<0.01, *** p<0.001, ****p<0.0001. Gray shaded areas represent the 95% CI for the control samples.

Immune phenotyping of the TME revealed significant changes following treatment with either ^177^Lu-NM600 or ^225^Ac-NM600 in murine Myc-CaP and TRAMP-C1 tumor models. Immune profiles were evaluated at days 7, 14, and 28 post-injection (p.i.) of either ^177^Lu-NM600 (5.55 or 18.5 MBq) or ^225^Ac-NM600 (7.4 or 18.5 kBq), with vehicle-treated animals serving as baseline controls. Results highlighted a complex, dose-dependent modulation of immune populations within the TME. Treatment with high dose of ^177^Lu-NM600 led to a significant increase in infiltrating immunosuppressive cells within TRAMP-C1 tumors by day 28, including myeloid-derived suppressor cells (MDSCs: CD45+CD11b+GR-1+) (p = 0.0003) and regulatory T cells (Tregs: CD4+CD25+FoxP3+) (p = 0.0005) (**Figures 1A, B**). In Myc-CaP tumors, MDSC levels similarly increased (p = 0.0008) (**Figure 1G**), whereas Treg levels decreased (**Figure 1H**) with both doses. Across all ^177^Lu-NM600-treated animals, CD8/Treg ratios fluctuated over the four-week study period but ultimately decreased in TRAMP-C1 and increased in Myc-CaP by day 28 compared to controls (**Figures 1C, I**). Conversely, ^225^Ac-NM600 treatment significantly reduced (p<0.05) both MDSCs and Tregs, leading to a steady increase in the CD8/Treg ratio (p = 0.0171 at day 28) in TRAMP-C1 mice receiving 18.5 kBq ^225^Ac-NM600 (**Figures 1D–F**). Changes in the CD8/Treg ratio were primarily driven by Treg depletion, as CD8+ T cell levels in TRAMP-C1 tumors remained largely unaffected by either ^177^Lu-NM600 or ^225^Ac-NM600 at all time points examined (**Supplementary Figures S2C, F**). Interestingly, in Myc-CaP tumors, ^225^Ac-NM600 treatment led to a significant increase in MDSCS (**Figure 1J**), a significant reduction in infiltrating Tregs (**Figure 1K**), yet CD8/Treg ratios remained unchanged (**Figure 1L**). Overall, RPT significantly reduced CD3+ and CD4+ T cells in both tumor models (**Supplementary Figure S2** **).**

### 3.2 Enhanced CTL function following ^225^Ac-NM600 treatment

**Figure 2:**
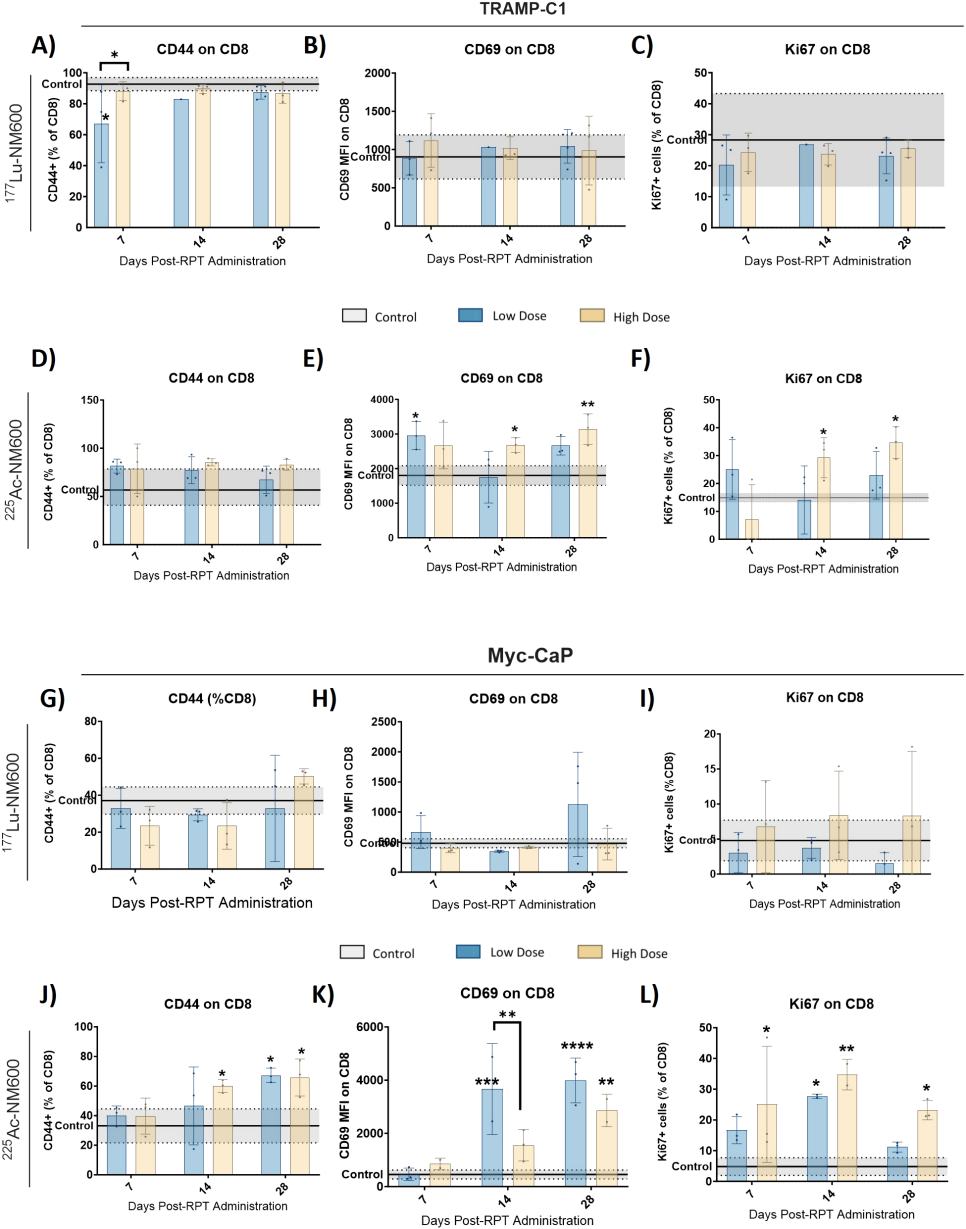
^225^Ac-NM600 promotes an active CD8+ repertoire with increased levels of memory, activation, and proliferation markers. Flow cytometry analysis of CD44+, CD69+, and Ki67+ in TRAMP-C1 tumor bearing mice receiving ^177^Lu-NM600 **(A-C)** or ^225^Ac-NM600 **(D-F)** and in Myc-CaP tumor bearing mice that received ^177^Lu- NM600 **(G-I)** or ^225^Ac-NM600 **(J-L)**. Data are presented as % of CD8+ T cells. Statistical analysis compares to controls or otherwise noted. *p<0.05, **p<0.01, ***p<0.001, ****p<0.0001.

**Figure 3:**
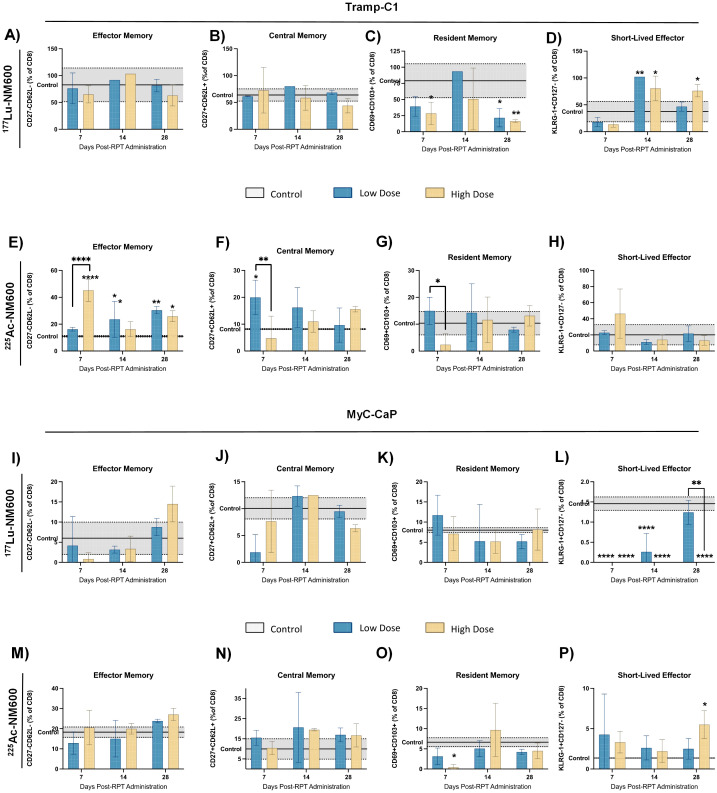
Expression levels of effector, central, resident and short-lived effector memory markers in TRAMP-C1 tumor bearing mice that received ^177^Lu-NM600 **(A-D)** or 225Ac-NM600 **(E-H)** and in Myc-CaP tumor bearing mice that received ^177^Lu-NM600 **(I-L)** or ^225^Ac-NM600 **(M-P)**. Statistical analysis when compared to controls or otherwise noted. **p<0.01, ***p<0.001, ****p<0.0001.

The functional status of CTLs was assessed using markers for activation and memory. In both tumor models, treatment with ^177^Lu-NM600 had minimal impact on CD44+ memory cell populations, CTL activation (CD69+), and proliferating cell markers (Ki67+) (**Figures 2A-C, G-I** **).** In contrast, ^225^Ac-NM600 significantly enhanced CD8+ T cell activation, as evidenced by increased expression of CD69 (p = 0.0042) and Ki67 (p = 0.0193) in TRAMP-C1 tumors (**Figures 2D-F** **),** and CD69 (p < 0.0001), Ki67 (p = 0.0235), and CD44 (p = 0.0152) in Myc-CaP tumors (**Figures 2J–L** **).** No significant changes were found for effector, central or resident memory in either animals treated with 177Lu-NM600 (**Figure 3A-C, I-K**). Effector memory CD8+ T cells (CD27-CD62L-) were significantly increased in TRAMP-C1 tumors treated with ^225^Ac-NM600 (p = 0.004) (**Figure 3E**), particularly at earlier time points, alongside modest increases in central memory (CD27+CD62L+) (**Figure 3F**) and resident memory (CD69+CD103+) markers (**Figure 3G**). In contrast, these memory subsets remained largely unaltered in Myc-CaP tumors (**Figures 3M–O**). Short-lived effector cells (KLRG-1+CD127+) exhibited differential responses: significant increases were observed in TRAMP-C1 tumors treated with ^177^Lu-NM600 (**Figure 3D**), while decreases were noted in Myc-CaP tumors (p < 0.0001) (**Figure 3L**). These cells were unaffected by ^225^Ac-NM600 in both tumor models, indicating selective modulation by radiation type and isotope (**Figures 3H, P**).

### 3.3 ^225^Ac-NM600 enhances PD-1 expression on CD8+ T cells and PD-L1 expression on myeloid cells

**Figure 4:**
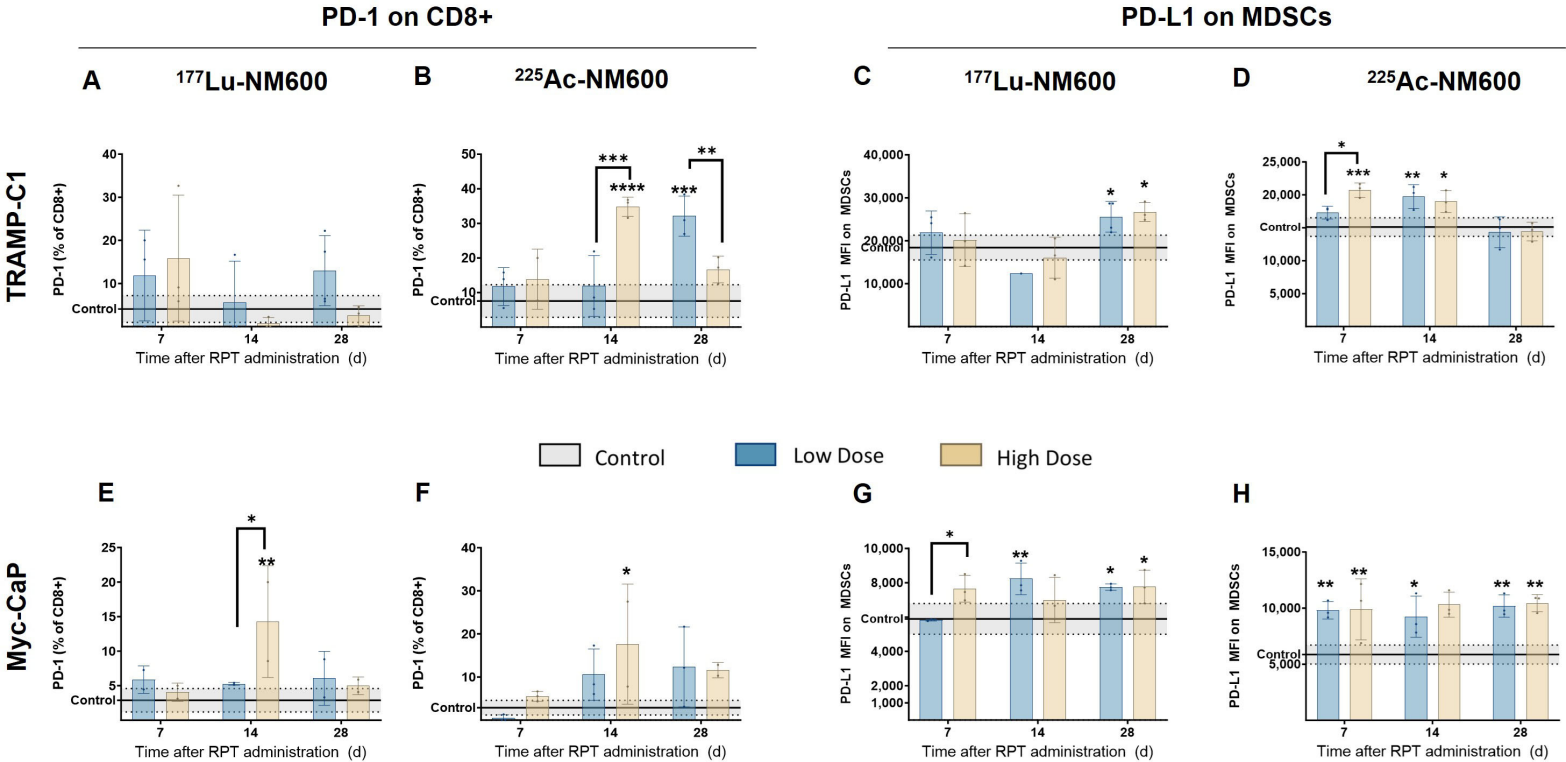
^225^Ac-NM600 increases PD-1 expression on CD8+ T cells and PD-L1 expression on MDSCs. PD-1 expression on CD8+ cells and PD-L1 expression on MDSCs after administration of ^225^Ac-NM600 or ^177^Lu-NM600 in TRAMP-C1 **(A-D)** and Myc-CaP **(E-H)** tumor-bearing mice. Statistical analysis compared to controls or otherwise noted. *p < 0.05, **p<0.01, ***p<0.001, ****p<0.0001.

TRAMP-C1 tumors exhibited no significant differences in PD-1 expression on CD8+ T cells among ^177^Lu-NM600 treatment groups (**Figure 4A**). In contrast, ^225^Ac-NM600 treatment resulted in a significant increase in PD-1 expression (p < 0.0001) on days 14 and 28 post-treatment (**Figure 4B**). Similarly, Myc-CaP mice showed elevated PD-1 expression (p < 0.05) on infiltrating CD8+ T cells following high injected activity of either RPT agent on day 14 (**Figures 4E, F**). Additionally, Myc-CaP mice demonstrated significantly increased PD-L1 expression on MDSCs (**Figures 4C, D, G, H**), with the highest levels observed in response to ^225^Ac-NM600 treatment, which resulted in a maximum expression level approximately twice that of controls (**Figure 4H**). Given that the combination of RPT and PD-1/PD-L1 inhibitors has been proposed as a potentially synergistic therapeutic strategy, we examined whether RPT influenced PD-L1 expression in tumor cells (**Supplementary Figure S3**). Notably, ^225^Ac-NM600 administration did not alter PD-L1 expression in TRAMP-C1 or Myc-CaP tumor cells. However, ^177^Lu-NM600 treatment led to a significant decrease (p < 0.01) in PD-L1 expression in TRAMP-C1 tumors compared to controls (**Supplementary Figure S3A**). These findings suggest that ^225^Ac-NM600 not only modulates immune cell populations but also enhances immune checkpoint marker expression, supporting the potential for combination therapies involving PD-1/PD-L1 inhibitors.

### 3.4 RPT elicits context-dependent changes in TME cytokine and chemokine profiles

**Figure 5:**
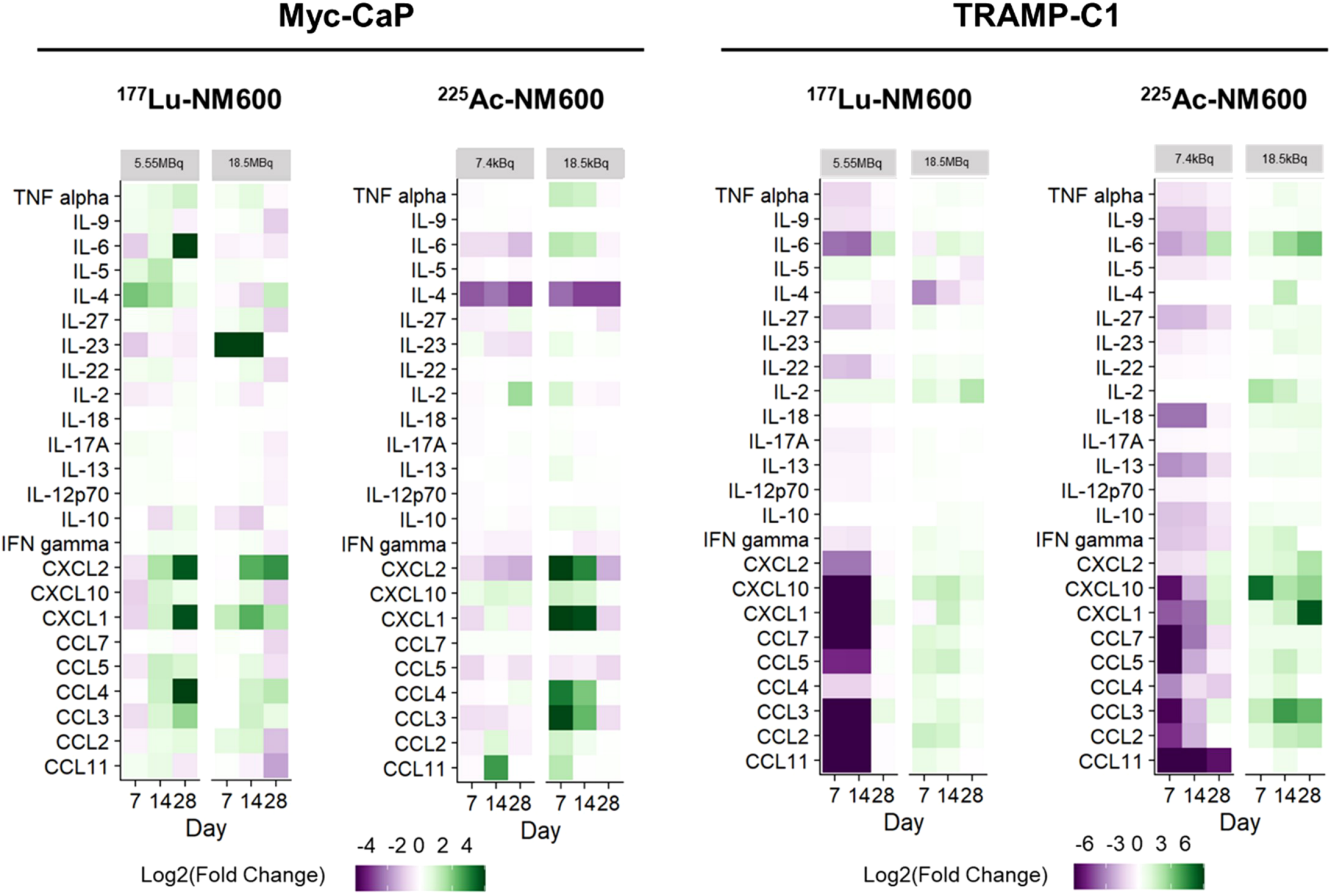
RPT-induced cytokine and chemokine changes in PCa TME. In TRAMP-C1, but not Myc-CaP tumors (n=3), 225Ac-NM600 (7.4 kBq) or 177Lu-NM600 (5.55MBq) decreased cytokine and chemokine concentrations while 225Ac-NM600 (18.5 kBq) or 177Lu-NM600 (18.5 MBq) increased their levels. In Myc-CaP tumors (n=3), some cytokines such as IL-4 and IL-23 increased with 177Lu-NM600 but decreased with 225Ac-NM600, while most chemokines significantly increased with an 18.5 kBq of 225Ac-NM600. Overall, concentration changes were more prominent with 225Ac-NM600 than 177Lu-NM600. Data is presented as Log2(Fold Change).

**Figure 6:**
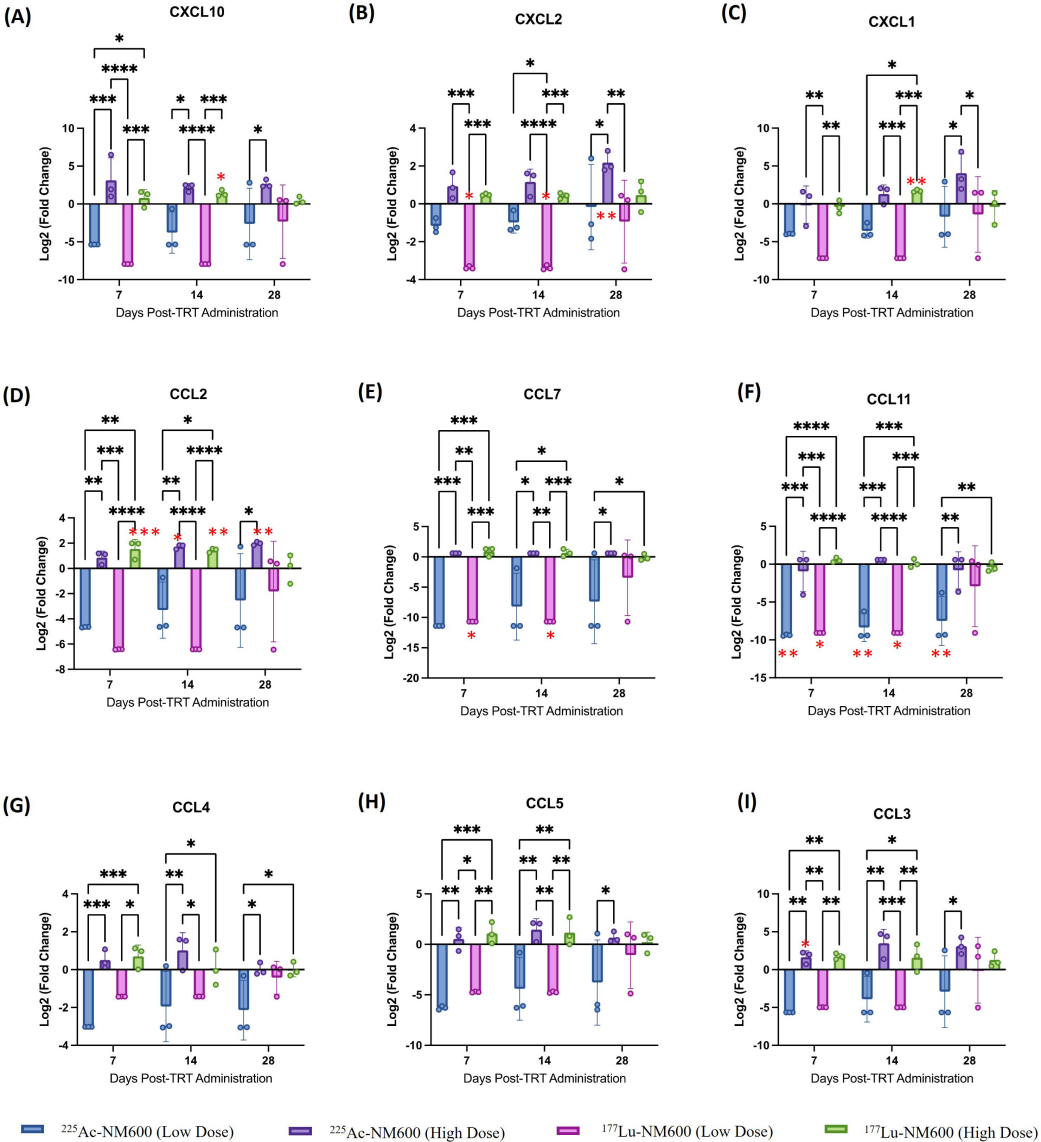
Analysis of **(A)** CXCL10, **(B)** CXCL2, **(C)** CXCL1, **(D)** CCL2, **(E)** CCL7, **(F)** CCL11, **(G)** CCL4, **(H)** CCL5, **(I)** CCL3 chemokines in TrampC-1 tumor bearing mice after administration of 5.55 MBq or 18.5 MBq of 177Lu-NM600 or 7.4 kBq or 18.5 kBq of 225Ac-NM600. **p<0.01, ***p<0.001, ****p<0.0001. * in red denote statistical significance when compared to controls.

To identify potential differences in cytokine profiles following treatment with either ^177^Lu-NM600 or ^225^Ac-NM600, we analyzed a panel of 26 immune cytokines and chemokines in tumor tissue collected at multiple time points (days 7, 14, and 28) post-RPT administration. **Figure 5** presents a heatmap of these findings, displayed as fold change relative to controls. Overall, lower injected activities of ^177^Lu-NM600 or ^225^Ac-NM600 resulted in reduced cytokine and chemokine levels in TRAMP-C1 tumors, while higher injected activities led to increased levels. The magnitude of these changes was greater for ^225^Ac-NM600 than for ^177^Lu-NM600. To better categorize these responses, analytes were grouped by function into Th1, Th2, Th17, and chemokine-associated signatures, revealing distinct patterns across different doses and isotopes (**Supplementary Figures S4–S6**). A statistical analysis comparing treatment arms with controls is summarized in **Supplementary Table S5**. In Myc-CaP tumors, proteins associated with Th1, Th2, and Th17 immune responses remained largely unchanged. However, in TRAMP-C1 tumors, these targets exhibited dose-dependent variations, with lower injected activities of either RPT agent significantly decreasing analyte levels, while higher doses had the opposite effect. A similar trend was observed for chemokine expression (**Figure 6**), where high injected activities resulted in significantly reduced levels of CXCL10, CXCL2, CXCL1, CCL2, CCL7, CCL3, CCL4, CCL5, and CCL11 compared to all other groups.

## 4 Discussion

Many studies in the field of radiation therapy have prioritized strategies to maximize the radiation dose delivered specifically to prostate tumor cells. These approaches typically follow the principle of administering the highest dose tolerable by normal tissues, often referred to as the “maximum tolerable dosing” paradigm. This methodology, however, rests on the assumption that tumors cannot be overdosed with radiation, an assumption that largely overlooks the complex and dynamic interactions within the TME (23). Overwhelming preclinical and clinical data indicate that antitumor effects of radiation are partly mediated by radiation-induced immunological effects (22, 24–27). The TME, which encompasses a variety of non-tumor cells, signaling molecules, and extracellular matrix components surrounding the tumor, plays a crucial role in tumor progression, resistance, and response to treatment. By neglecting this context, conventional strategies may fail to optimize therapeutic outcomes or fully address the multifaceted nature of tumor biology. Emerging evidence from a wealth of preclinical and clinical studies has shifted this perspective, highlighting that the antitumor effects of radiation are not solely due to direct cytotoxicity to tumor cells. Instead, these effects are partially mediated through radiation-induced immunological responses. Ionizing radiation has been shown to influence immune processes by enhancing tumor antigen presentation, promoting pro-inflammatory signaling, and facilitating immune cell infiltration into the tumor site. These immunological mechanisms contribute significantly to the overall therapeutic efficacy of radiation therapy, suggesting that a deeper understanding of these processes could unlock new avenues for optimizing treatment.

Despite these advances, much of our understanding of the radiobiology underlying targeted radiation therapy, such as radiopharmaceutical therapy (RPT), has been derived from preclinical studies conducted in immunocompromised animal models. While these models provide valuable insights, they lack the critical immunological components necessary to fully appreciate the interplay between radiation and the immune system. Consequently, the absence of this immunological dimension limits the translational applicability of these findings to human systems, where the immune response is integral to therapeutic outcomes. Furthermore, RPT studies have rarely leveraged a theranostic approach to enable dosimetry calculations; therefore, knowledge gaps exist surrounding the absorbed dose and radioisotope dependencies of RPT immunomodulation and toxicity. Additionally, RPT studies have seldom incorporated a theranostic approach, which integrates diagnostic imaging and therapeutic intervention to enable personalized dosimetry. Dosimetry calculations are essential for determining the absorbed radiation dose delivered to both tumor and normal tissues, which in turn informs treatment planning and minimizes toxicity. Without this approach, significant gaps persist in our understanding of how absorbed dose and specific radioisotopes influence RPT’s immunomodulatory effects and associated toxicities. Addressing these gaps is vital for advancing the field and realizing the full potential of RPT as a modality that synergizes direct tumor targeting with immune activation (24–26, 28, 29).

Since the effects of alpha-emitting RPT agents in the tumor microenvironment of PCa are relatively unknown, we aimed to investigate how different emissions and absorbed doses can elicit immunological changes in the TME of two syngeneic PCa models. ^177^Lu-NM600 biodistribution and voxel-based Monte Carlo estimations of tumor and relevant normal tissues dosimetry was used through an in-house platform (RAPID) (27). This permitted absorbed dose-based comparisons between the different agents and dosing regimens, essential to dissecting the mechanistic underpinnings of RPT immunomodulation. Additional investigations are necessary to fully unravel the molecular basis of the enhanced efficacy observed with ^225^Ac-NM600. However, our results showed evidence of dose and tumor model-specific immunological changes in the irradiated TME after treatment with ^225^Ac-NM600, which could have contributed to the enhanced antitumor response observed. Cellular and secretory immunological responses within the TME can directly relate to and dictate treatment outcomes in PCa (30, 31). Among the cellular components, infiltrating CD8+ CTLs are the primary effectors of tumor cell lysis through granule and cytokines release; with higher levels of CTLs infiltration often correlating with greater antitumor effects (32, 33). However, increased CTL infiltration in the PCa TME does not always correlate with better outcomes (34, 35), mainly because CTLs are effectively neutralized by anti-inflammatory and immunosuppressive cell lineages such as MDSCs and Tregs (36, 37). Thus, the ratio of infiltrating CTLs to suppressive cells constitutes a better metric of the immunological status of the TME (38), with a decreased CD8/Treg ratio being generally recognized as a negative prognosticator in solid tumors (39–41). Notably, Lin et al. (27) reported similar findings where EBRT led to significant MDSC and Treg infiltration that hindered antitumor immunity. These results indicated the inability of low linear energy transfer (LET) radiation, both β– and X-rays, to eradicate relative radioresistance immunosuppressive cell lineages effectively (26, 42, 43), resulting in relative enrichments in infiltrating Tregs and MDSCs following irradiation. The observed increase in immunosuppressive cell populations like MDSCs and Tregs with ^177^Lu-NM600 treatment may have contributed to the reduced CD8+ T cell to regulatory cell ratios compared to baseline. We have recently shown similar results with another β– emitter, ^90^Y-NM600, where the accumulation of Tregs within the TME negated the antitumor response to ^90^Y-NM600 and led to detrimental effects in combination with anti-PD1 immunotherapy (44). Tumor control was then rescued through Treg depletion using an anti-CTLA4 antibody, evidencing the significant impact of Tregs on the tumor radiobiology of these prostate tumors.

Moreover, treatment with ^225^Ac-NM600 demonstrated significant immunomodulatory effects, particularly in depleting suppressive immune lineages, which contributed to a higher CD8/Treg ratio and enhanced antitumor efficacy compared to ^177^Lu-NM600. This outcome is noteworthy, as the ratio of cytotoxic CD8+ T cells to Tregs is a critical determinant of effective anti-tumor immunity. While ^225^Ac-NM600 caused a modest reduction in CTL infiltration, it nevertheless induced an overall pro-inflammatory immune cell balance within the TME. These findings underscore previously unrecognized advantages of ^225^Ac high-linear energy transfer radiation in mitigating immunosuppressive cell populations in prostate cancer (PCa), a disease often characterized by a relatively immunologically “cold” phenotype (45–47). Beyond the depletion of suppressive populations, other cellular and molecular effects may also contribute to the immunological profile of PCa. For example, factors such as CTL senescence and anergy, which impair the functionality of cytotoxic T cells, could be contributing to the immune evasion mechanisms observed in PCa. Importantly, treatment with ^225^Ac-NM600 induced an upregulation of activation and proliferation markers, including CD44+, CD69+, and Ki67+, on CD8+ T cells. The increased expression of these markers suggests a potentially more active and proliferative T cell repertoire, potentially transforming the TME into a more “hot” or immunologically active state. However, while these markers are indicative of T cell activation and proliferation, they represent only a partial view of the complex landscape of anti-tumor immunity.

It is crucial to acknowledge that the presence of activation and proliferation markers does not necessarily translate into an effective anti-tumor immune response. One of the major barriers to such responses is the state of T cell exhaustion, a phenomenon characterized by diminished effector functions and the expression of inhibitory receptors. T cell exhaustion, often observed in chronic inflammatory environments such as the TME, represents a significant hurdle to achieving sustained anti-tumor activity. Consequently, further investigation is required to evaluate the functional implications of the observed increases in activation and proliferation markers on CD8+ T cells after ^225^Ac-NM600 treatment. Specifically, it is essential to determine whether these changes correlate with genuine enhancements in anti-tumor activity or whether they coexist with signs of exhaustion that may limit their overall efficacy. Interestingly, upregulation of both CD69 and PD-1 was observed on tumor-infiltrating CD8+ T cells following both beta-emitting and alpha-emitting radiotherapies, in contrast to untreated tumors. While these findings could indicate differential patterns of T cell activation or inhibition, they also raise critical questions regarding the functional state of these T cells. To fully elucidate the role of T cell responses in the context of ^225^Ac-NM600 therapy, it is essential to assess the expression of additional inhibitory receptors such as CTLA-4, LAG-3, and TIM-3. Moreover, functional assays to evaluate the ability of T cells to produce key effector cytokines, such as IFN-γ and TNF-α, would provide a more comprehensive understanding of their capacity to mediate effective anti-tumor immunity within the TME. Such investigations will be instrumental in clarifying whether the observed immune alterations truly reflect a reinvigorated anti-tumor immune response or if they represent a complex interplay between activation, proliferation, and exhaustion states in the context of radiation-induced immunomodulation.

^225^Ac-NM600 elicited notable cellular immunomodulatory effects, particularly through the depletion of Tregs and myeloid-derived suppressor cells (MDSCs), which resulted in an increased CD8+/Treg-MDSC ratio in TRAMP-C1 tumor-bearing mice. This is a significant finding, as recent evidence has demonstrated that Treg depletion is critical for improving the antitumor efficacy of radiopharmaceutical therapy (RPT). Consequently, the ability of ^225^Ac-NM600 to reverse immunosuppressive traits within the TME likely represents a key immunomodulatory mechanism potentially driving anti-tumor efficacy. Nevertheless, further studies are needed to determine whether this efficacy is predominantly mediated by pro-inflammatory effects, anti-immunosuppressive effects, or a synergistic combination of both. In addition to its cytotoxic effects on tumor cells, ^225^Ac-NM600 demonstrated the capacity to induce immune modifications within irradiated tumors, underscoring its potential role in enhancing antitumor immunity. To further elucidate these mechanisms, additional research is required to explore the intricate interplay between radiation-induced cell death and antitumor immune responses. Expanding our understanding of alpha-particle RPT immunomodulation will be critical for optimizing its therapeutic potential and integrating it into broader immunotherapy strategies. In addition to cellular components, mediators such as cytokines and chemokines play critical roles in shaping the pro- or antitumor properties of the TME. Using a Luminex-based multiplex assay, we observed distinct patterns in cytokine and chemokine expression in TRAMP-C1 tumors depending on the absorbed dose delivered by the radiopharmaceutical therapy of the radiopharmaceutical therapy. At lower IAs, both pro-inflammatory and suppressive cytokines/chemokines were reduced, suggesting a general dampening of immune activity. In contrast, higher doses led to an increase in both pro-inflammatory and suppressive mediators. Despite this dual effect, high doses were associated with superior antitumor responses compared to low doses. This indicates that the increase in pro-inflammatory cytokines likely plays a dominant role in driving antitumor efficacy, although this conclusion is complicated by the concurrent effects of increased direct tumor cell killing at higher IAs. To disentangle these overlapping mechanisms, further research is necessary to clarify the roles of individual cytokines and chemokines in mediating the observed antitumor effects. Specifically, studies investigating the timing of cytokine and chemokine release and their relative contributions to immune activation versus suppression within the TME will be critical. Such insights could help refine therapeutic strategies to optimize the balance of pro-inflammatory mediators while minimizing the impact of suppressive signals, ultimately enhancing the efficacy of radiopharmaceutical therapies (42).

Altogether, compared to ^177^Lu-NM600, ^225^Ac-NM600 demonstrated markedly stronger pro-inflammatory immunomodulatory activity. This included elevated activation markers on CTLs, depletion of regulatory immune cell populations (Tregs and MDSCs), and an increase in Th1-biased cytokine profiles, all of which likely contributed to the superior antitumor efficacy of ^225^Ac-NM600. Importantly, differential antitumor and immunological responses were observed between the two prostate cancer (PCa) models investigated, highlighting the complex interplay of factors influencing therapeutic outcomes. Key variables, such as mutational burden, cytokine and chemokine milieu, leukocyte infiltration, and immune checkpoint molecule expression, can vary significantly across tumor models and shape their immune susceptibility. Furthermore, TRAMP-C1 and Myc-CaP models differ in several immunological and biological characteristics, including androgen receptor (AR) sensitivity, antigen presentation capabilities, interferon (IFN) responsiveness, and baseline lymphocyte infiltration. These distinctions contribute to their varied patterns of immunomodulation (43, 48–50). Our findings emphasize the importance of employing multiple preclinical models to evaluate immunological mechanisms in radiopharmaceutical therapy. Given the inherent variability across tumor types, drawing broad conclusions from a single model is challenging. Expanding the scope of model systems enhances the robustness of conclusions and provides a more comprehensive understanding of the diverse factors that shape immune responses to therapy.

The increased expression of PD-1 on CD8+ T cells and PD-L1 on MDSCs observed following ^225^Ac-NM600 (RPT) suggests a potential synergy with anti-PD-1 inhibitors to further enhance antitumor efficacy. Future studies will explore the therapeutic benefits of combining ICIs with alpha-particle RPT for the treatment of prostate cancer (PCa). Notably, previous investigations demonstrated that combining ^90^Y-NM600 RPT with anti-PD-1 ICI did not yield significant benefits over RPT alone in these models unless Tregs were concurrently depleted. While our current study highlights the potential for combining ICIs with RPT, it also acknowledges a limitation in the direct evaluation of PD-L1 expression on Tregs. Understanding the role of PD-L1 on Tregs within the TME is particularly relevant, as it could further elucidate mechanisms of immune evasion and guide synergistic strategies for RPT and ICI combinations. This area of investigation is especially pertinent given that one of the key immunomodulatory effects of ^225^Ac-NM600 is the depletion of Tregs. Consequently, future studies will prioritize this line of inquiry to assess the potential for ICI and alpha-RPT combinations in treating PCa. Although the presented results provide compelling evidence for enhanced immunomodulation with ^225^Ac-NM600, further validation in additional confirmatory models is essential to substantiate these findings. Identifying predictive biomarkers will also be critical to optimizing therapeutic strategies. It is important to note the inherent variability in *in vivo* immunophenotyping assays, which can obscure subtle changes and necessitate larger sample sizes for robust conclusions. Despite these limitations, our data establishes a foundational understanding of the distinct immunomodulatory properties of ^225^Ac and ^177^Lu radionuclides. In summary, this study underscores the complexity of radionuclide-induced immunological effects and the need for comprehensive, mechanistic investigations in well-powered, immunocompetent mouse models. Such efforts are essential to uncover both the potential synergies and possible counterproductive outcomes of combination therapies involving RPT and ICIs, paving the way for more effective therapeutic strategies.

Despite the promising results, several limitations must be considered when interpreting the findings. The sample size and study duration posed constraints, as the number of animals per cohort was limited by ethical and logistical considerations; larger sample sizes in future, more focused, studies would enhance statistical power and strengthen the robustness of findings. Tumor model selection also presents a limitation, as while the TRAMP-C1 and Myc-CaP models were chosen to represent distinct subtypes of advanced prostate cancer, the heterogeneity in PCa biology suggests the necessity for further validation in additional preclinical models, including patient-derived xenografts (PDXs) and genetically engineered mouse models (GEMMs), to ensure broader clinical applicability. Another critical consideration is dosimetry and toxicity, as while dosimetric analyses confirmed favorable tumor-to-normal tissue radiation dose ratios, the higher liver uptake of ^225^Ac-NM600 raises concerns about potential off-target effects; further optimization of dosing regimens and radiolabeling stability is required to minimize toxicity. The higher liver uptake associated with ^225^Ac-NM600 underscores a critical consideration in alpha-emitting radiopharmaceutical therapy: while alpha particles offer superior therapeutic efficacy due to their high linear energy transfer (LET), the redistribution of daughter nuclides can pose risks of off-target toxicity. Strategies to enhance daughter retention, such as improved chelation or encapsulation techniques, may mitigate these effects and optimize therapeutic outcomes Additionally, toxicity studies are of extreme importance in order to define the therapeutic window and understand implications of differences in alpha vs beta labeled probe biodistribution. Those studies are being carried out at the moment and will be published separately. Finally, longitudinal immune profiling remains an area for improvement, as immune phenotyping at multiple time points revealed dynamic changes in the TME; however, future studies should incorporate single-cell RNA sequencing and multiplexed spatial analysis to provide a more comprehensive characterization of the immune landscape and radiation-induced transcriptomic shifts in tumors.

Building on these findings, several key areas warrant further investigation. Combination strategies should be explored through preclinical evaluation of ^225^Ac-NM600 in combination with anti-PD-1/PD-L1 therapy in murine models to determine optimal sequencing and dosing for enhanced therapeutic synergy. Personalized theranostics represents another crucial area, where the integration of SPECT/CT-based dosimetry with tumor-specific immune profiling could enable the development of patient-specific dosing algorithms to maximize efficacy while minimizing toxicity. Additionally, expanded tumor models are necessary to improve translational relevance, requiring validation in humanized mouse models and patient-derived organoids that better reflect the heterogeneity observed in clinical prostate cancer cases. Finally, biomarker development remains essential for advancing precision medicine approaches by identifying predictive biomarkers of radiation-induced immune responses, facilitating stratified patient selection in future clinical trials and optimizing therapeutic outcomes.

## 5 Conclusion

We demonstrated that ^225^Ac-NM600 offers superior antitumor efficacy and immunomodulatory benefits compared to ^177^Lu-NM600 in advanced prostate cancer (PCa). By depleting regulatory T cells and myeloid-derived suppressor cells (MDSCs), while enhancing CD8/Treg ratios and cytotoxic T lymphocyte activation, we showed how ^225^Ac-NM600 transforms the tumor microenvironment into a more immunologically active state. Our findings demonstrated that ^225^Ac-NM600 elicited a stronger immunologically driven antitumor effect than its low LET counterpart, demonstrating ^225^Ac-NM600 therapeutic potential in PCa. These findings highlight its potential as a cornerstone for combination therapies with immune checkpoint inhibitors. Although more research is needed to fully elucidate the molecular mechanisms underlying the enhanced immune efficacy of ^225^Ac-NM600, this work expands our radiobiological insights regarding the radiation-type dependency of the immunomodulatory effects of RPT and provides a strong rationale for translating α-emitting RPT agents as single agents or in combination with immunotherapies in PCa. Our work emphasizes the importance of tailoring therapies to tumor-specific factors and paves the way for further validation in diverse models. By advancing targeted alpha therapies (TAT) like ^225^Ac-NM600, we aim to redefine treatment strategies for metastatic castration-resistant prostate cancer (mCRPC) and address an urgent clinical need.

## Data Availability

The raw data supporting the conclusions of this article will be made available by the authors, without undue reservation.
