# Supplementary material for: Immunomodulatory effects of alpha vs beta radiopharmaceutical therapy in murine prostate cancer
Source: Front Immunol. 2025 May 22;16:1563387. doi: 10.3389/fimmu.2025.1563387 (PMC12137342; doi:10.3389/fimmu.2025.1563387)
Supplement: Supplementary file 1 [file DataSheet1.docx]

Supplementary Material

Immunomodulatory Effects of Alpha vs Beta Radiopharmaceutical Therapy in Murine Prostate Cancer

**Carolina A. Ferreira^1^, Hemanth K. Potluri^2^, Mojdeh Mahmoudian^3^, Christopher Massey^4^, Joseph J. Grudzinski^4^, Amanda Carston^4^, Nathan Clemons^4^, Malick Bio Idrissou^4^, Anna Thickens^4^, Zachary Rosenkrans^4^, Cynthia Choi^5^, Caroline Kerr^6^, Anatoly Pinchuk^4^, Ohyun Kwon^4^, Justin J. Jeffery^7^, Bryan Bednarz^4,5^, Zachary Morris^6,7^, Jamey Weichert^2,4,7^, Douglas G. McNeel^2,7^, Reinier Hernandez ^4,5,6**^**

^1^ Departments of Radiology, Pharmacology and Toxicology and Biomedical Engineering, Michigan State University, East Lansing, MI, USA

^2^Department of Medicine, University of Wisconsin-Madison, Madison, WI, USA

^3^Department of Cell and Molecular Biology, Michigan State University, East Lansing, MI, USA

^4^Departments of Medical Physics and Radiology, University of Wisconsin-Madison, Madison, WI, USA

^5^Pharmaceutical Sciences Program, University of Wisconsin-Madison, Madison, WI, USA

^6^Department of Human Oncology, University of Wisconsin-Madison, Madison, WI, USA

^7^Carbone Cancer Center, University of Wisconsin-Madison, Madison, WI, USA

*** Correspondence:**

Reinier Hernandez
[hernandez6@wisc.edu](mailto:hernandez6@wisc.edu)

## Supplementary Figures

##
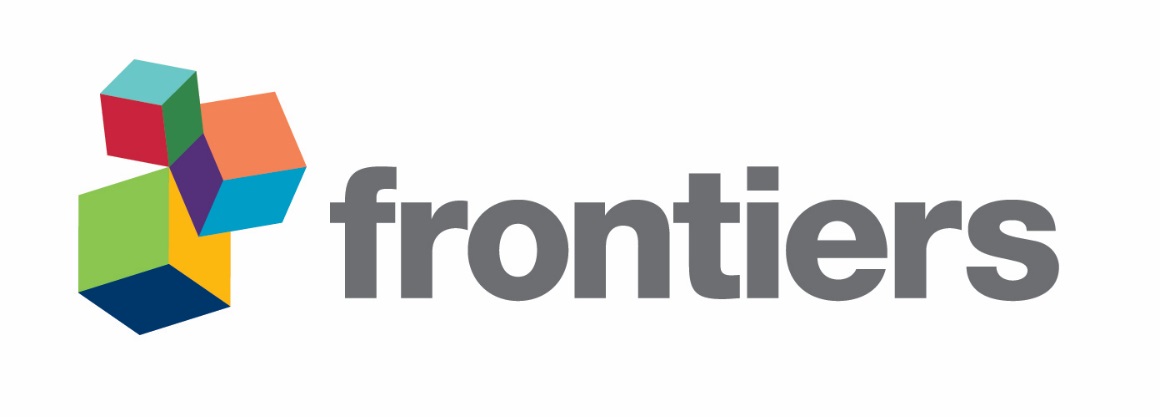


**Supplementary Table 1.** Summary of results from the dosimetry estimation (RBE = 1) for ^177^Lu-NM600 using *ex vivo* Biodistribution data.


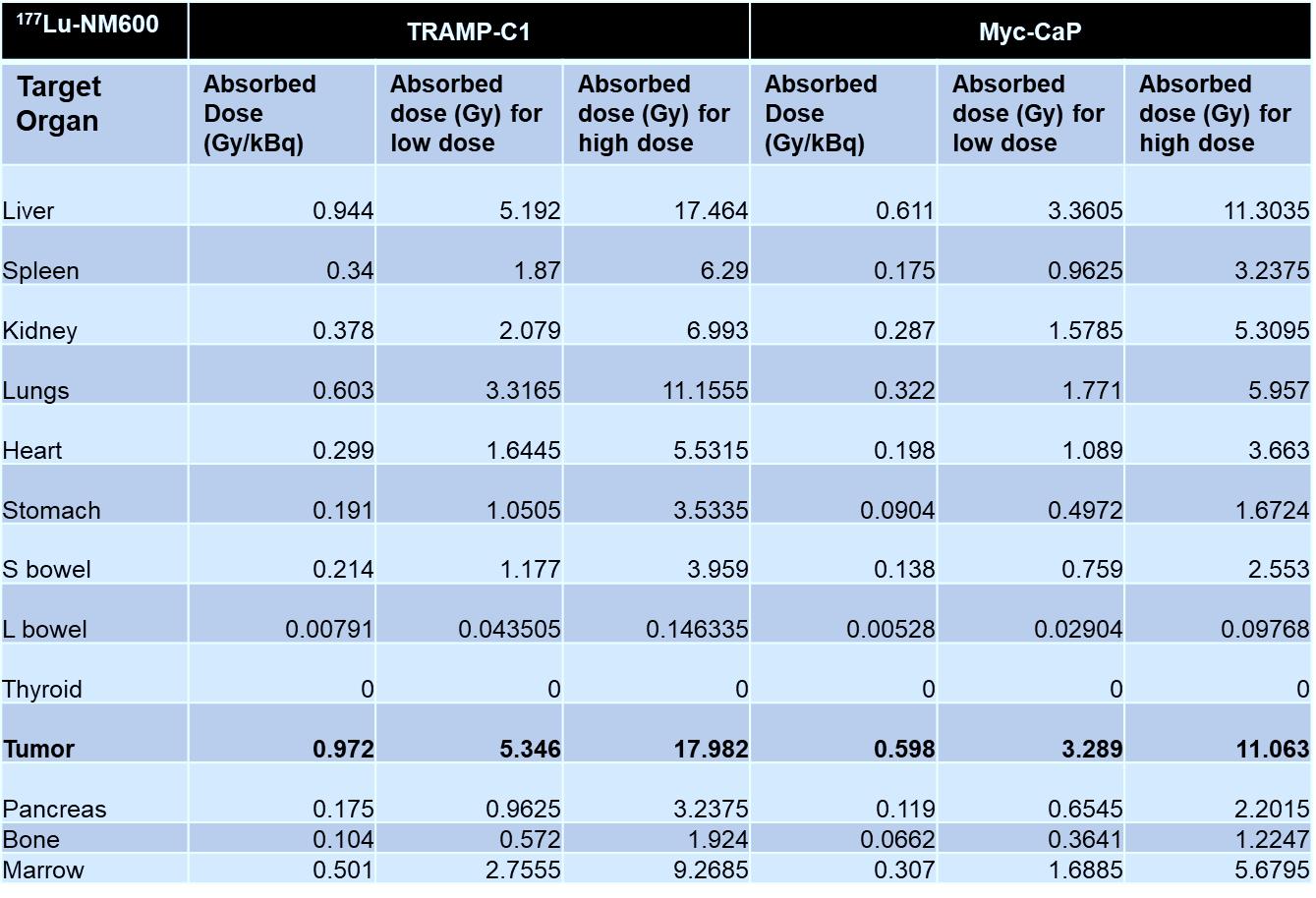


**Supplementary Table 2.** Summary of results from the dosimetry estimation for ^225^Ac-NM600 (RBE = 1) using *ex vivo* Biodistribution data.


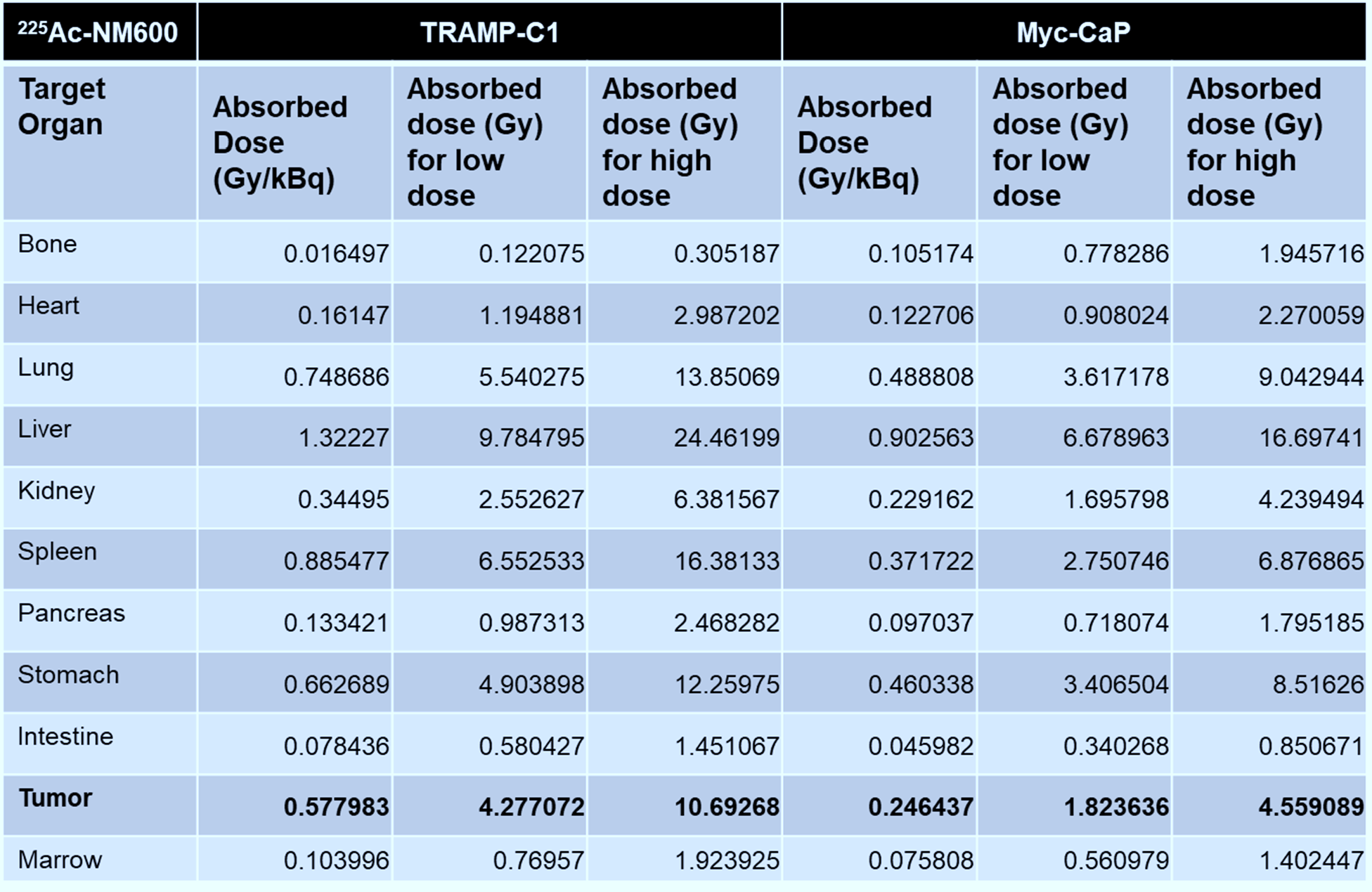


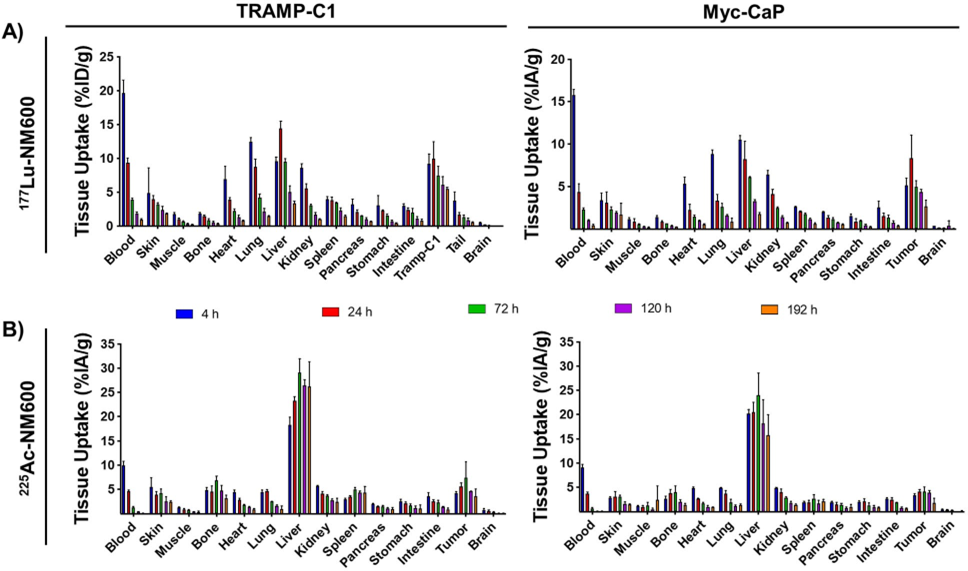


**Supplementary Figure 1**. *Ex vivo* biodistribution studies (n=3) were conducted in TRAMP-C1 and Myc-CaP tumor-bearing mice following intravenous administration of (A) 3.7 MBq of ^177^Lu-NM600 or (B) 7.4 kBq of ^225^Ac-NM600. The results showed comparable patterns of tumor uptake and normal tissue distribution for both radiopharmaceuticals, confirming their high tumor accumulation, prolonged retention, and hepatobiliary clearance as observed in SPECT/CT imaging. Data are reported as %IA/g (mean ± SD).


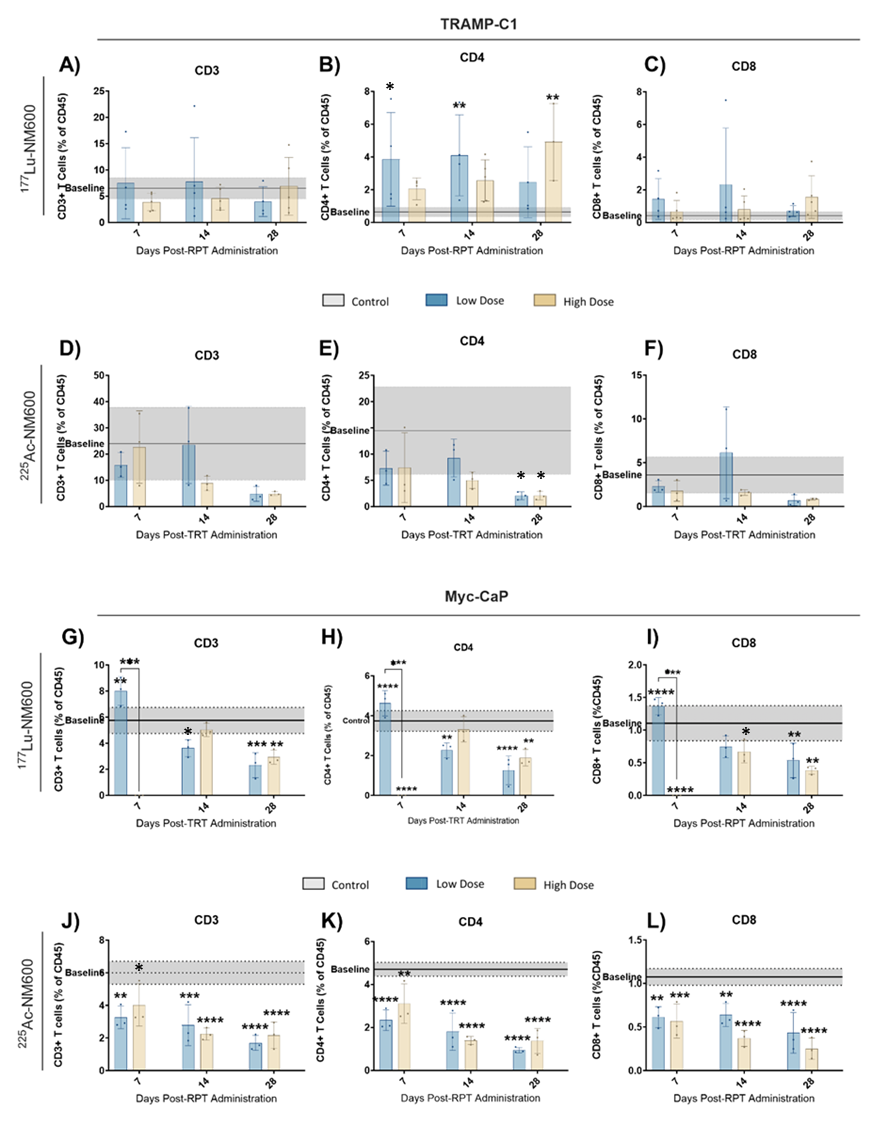


**Supplementary Figure 2.** Flow Cytometry analysis of CD3, CD4, and CD8 markers in TRAMP-C1 tumor bearing mice receiving ^177^Lu-NM600 **(A-C)** or ^225^Ac-NM600 **(D-F)** and in Myc-CaP tumor bearing mice that received ^177^Lu- NM600 **(G-I)** or ^225^Ac-NM600 **(J-L)**. Statistical analysis compares to controls or otherwise noted. *p < 0.05, ** p<0.01, *** p<0.001, ****p<0.0001.


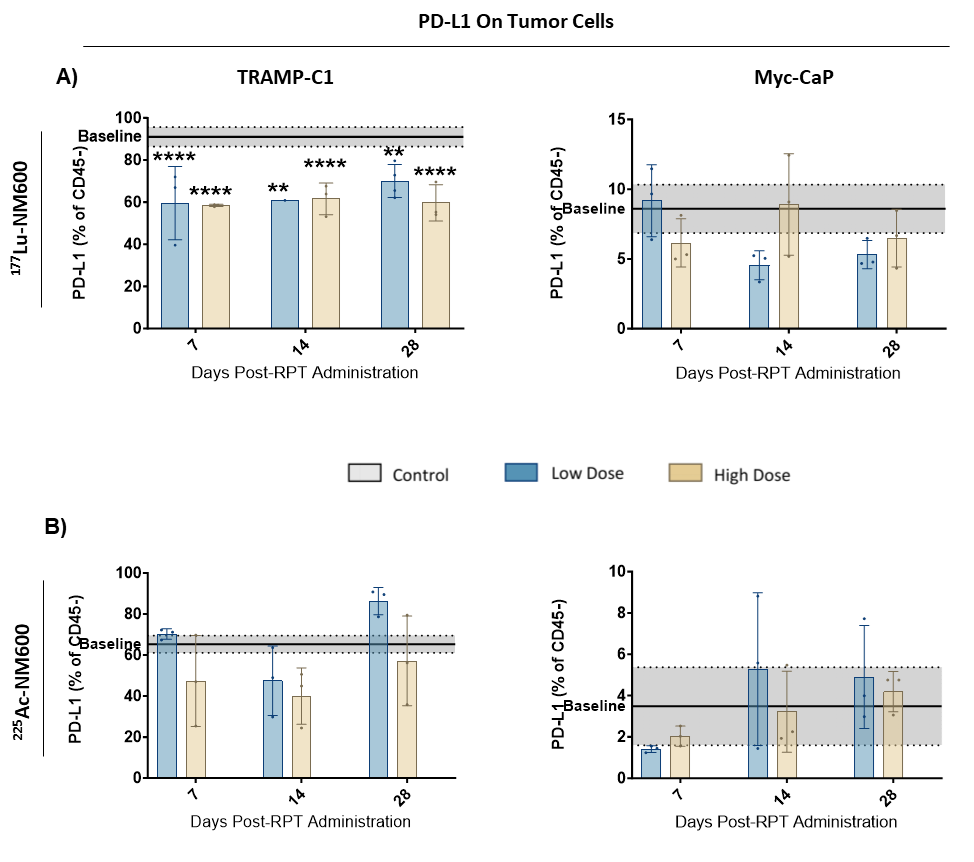


**Supplementary Figure 3.** Flow Cytometry analysis of PD-L1 expression levels (expressed as percentage of CD45-) on TRAMP-C1 and Myc-CaP tumors after administration of 177Lu-NM600 **(A)** or 225Ac-NM600 **(B)**. Statistical analysis compared to controls or otherwise noted. ** p<0.01, *** p<0.001, ****p<0.0001.


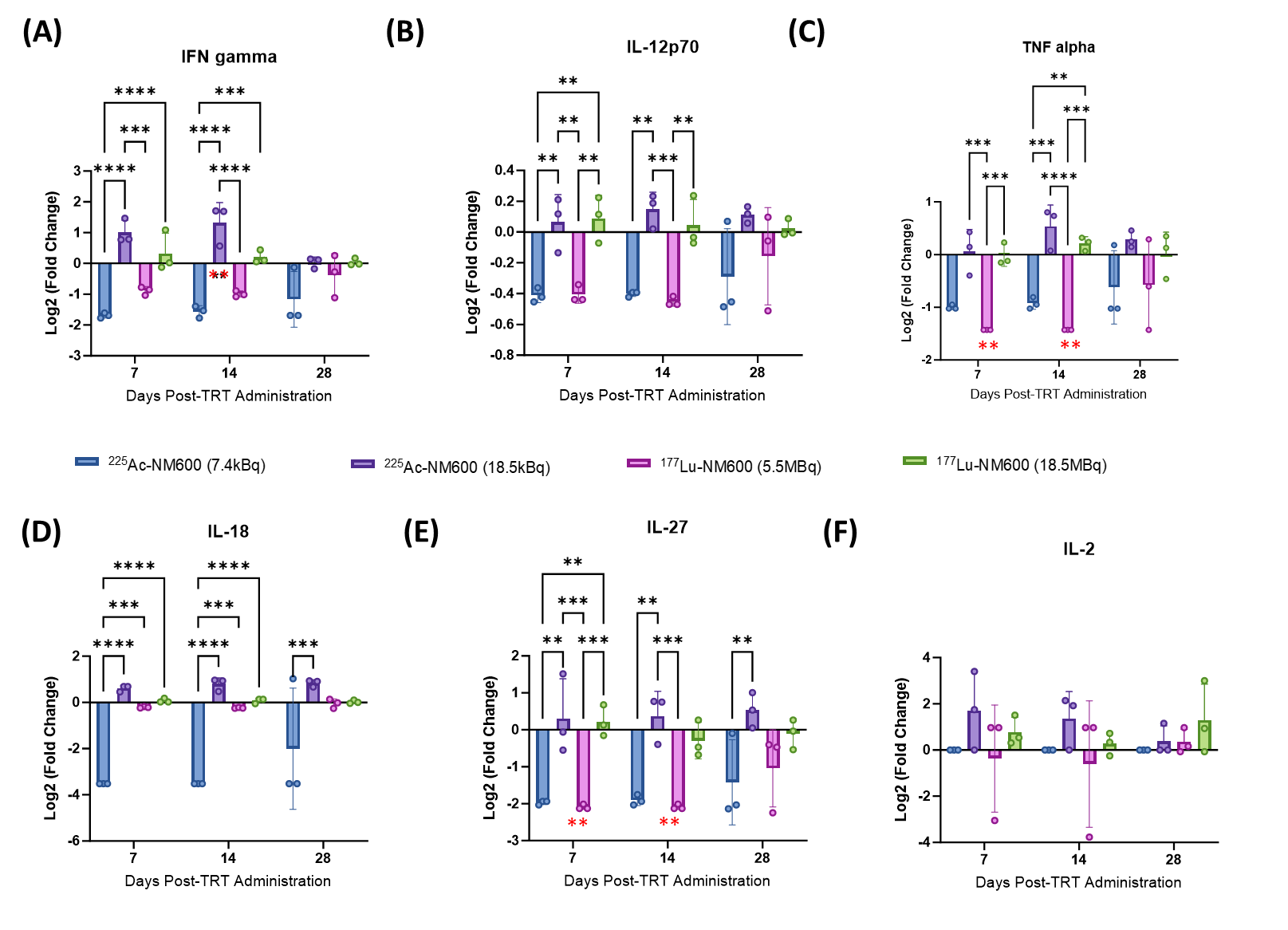


**Supplementary Figure 4.** Analysis of the Th1 phenotype cytokines **(A)** IFN gamma, **(B)** IL-12p70, **(C)** TNF alpha, **(D)** IL- 18, **(E)** IL-27, **(F)** IL-2 in TRAMP-C1 tumor bearing mice after administration of 5.55 MBq or 18.5 MBq of 177Lu-NM600 or 7.4 kBq or 18.5 kBq of 225AcNM600. ** p<0.01, *** p<0.001, ****p<0.0001. * in red denote statistical significance when compared to controls.


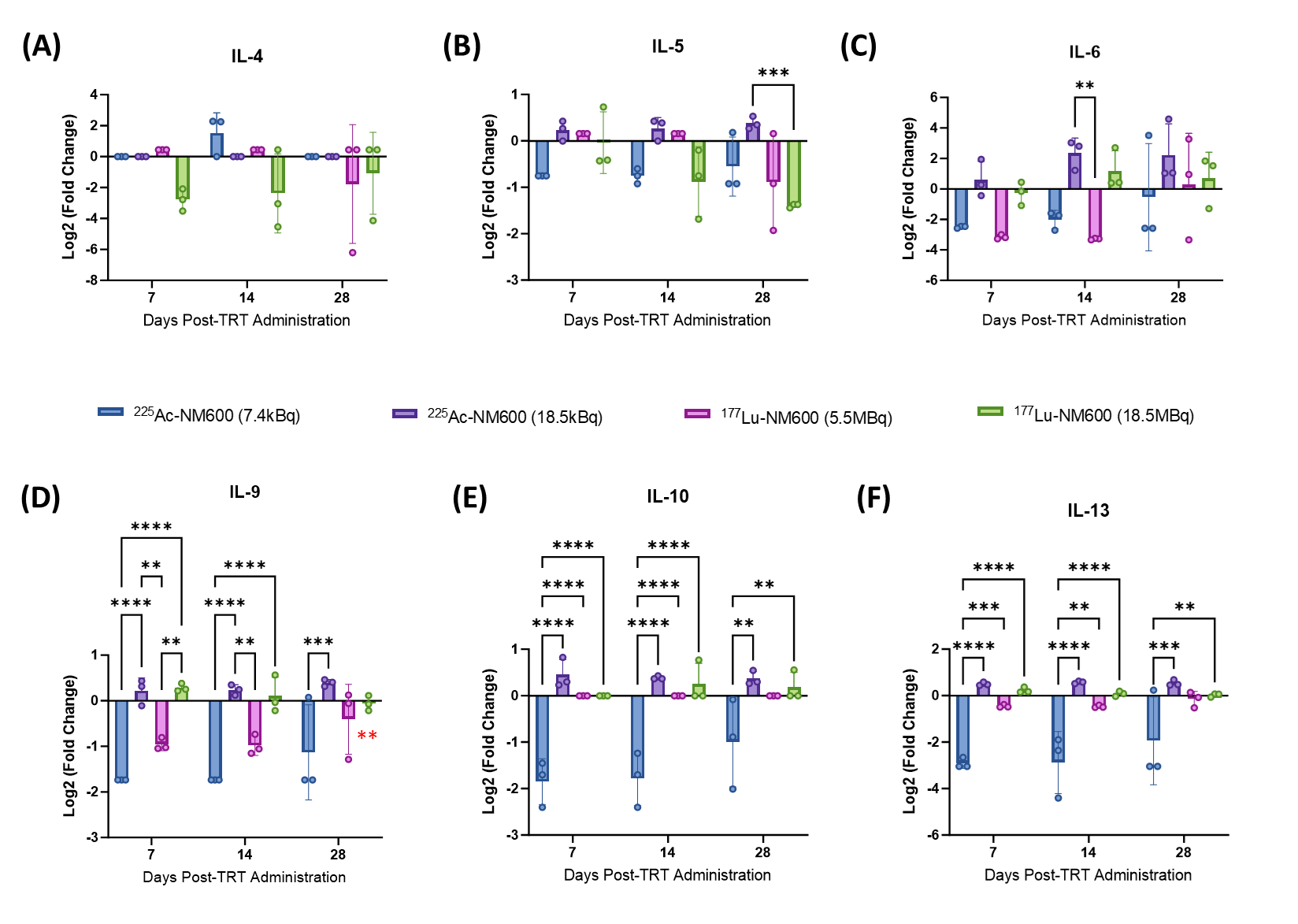


**Supplementary Figure 5.** Analysis of the Th2 phenotype cytokines (A) IL-4, (B) IL-5, (C) IL-6, (D) IL-9, (E) IL-10 and (F) IL-13 in TRAMP-C1 tumor bearing mice after administration of 5.55 MBq or 18.5 MBq of 177Lu-NM600 or 7.4 kBq or 18.5 kBq of 225Ac-NM600. ** p<0.01, *** p<0.001, ****p<0.0001. * in red denote statistical significance when compared to controls.


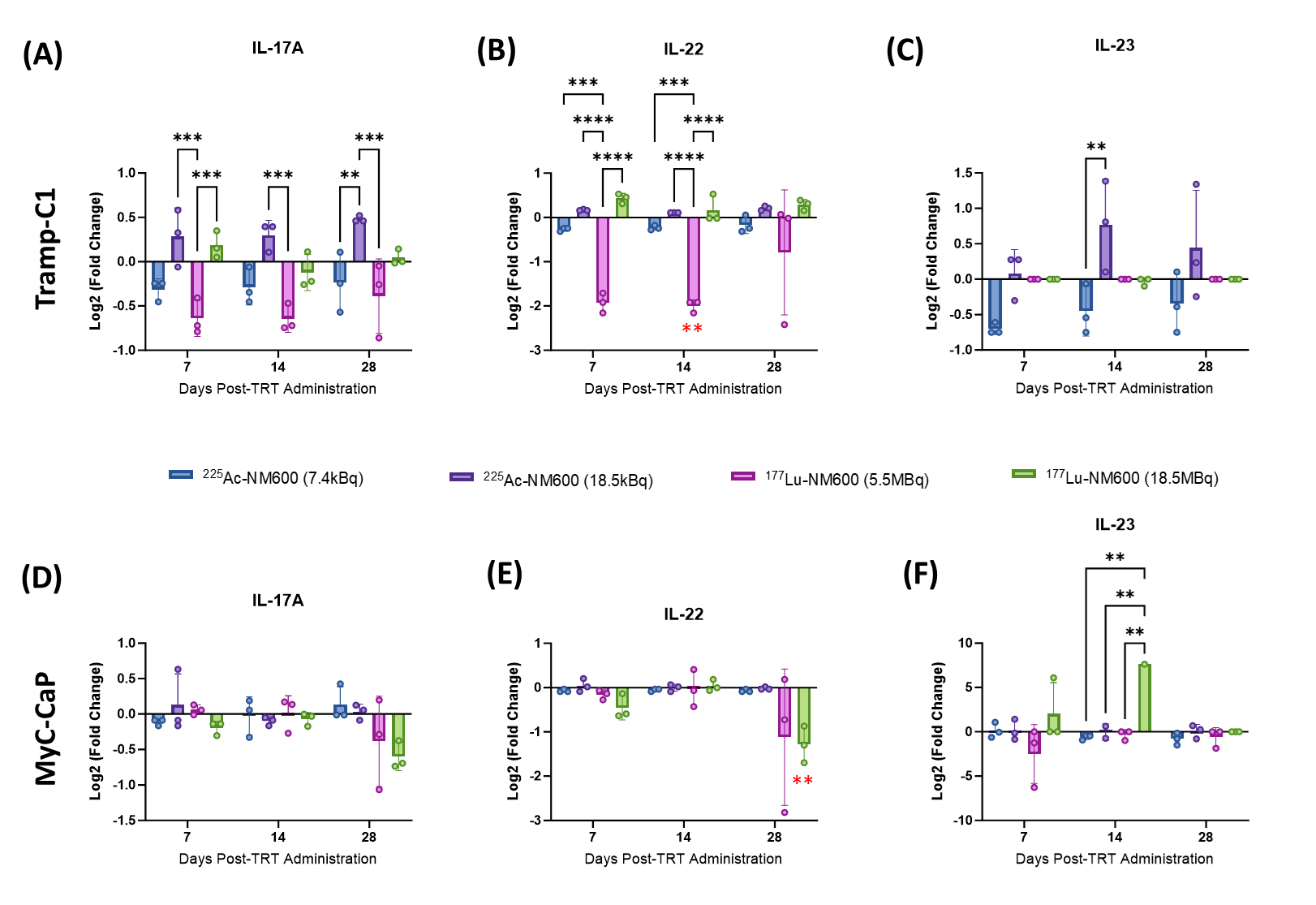


**Supplementary Figure 6.** Analysis of the Th17 phenotype cytokines **(A)** IL-17-A, **(B)** IL-22 and **(C)** IL-23 in TRAMP-C1 tumor bearing mice and **(D)** IL-17A, **(E)** IL-22, **(F)** IL-23 after administration of 5.55 MBq or 18.5 MBq of 177Lu-NM600 or 7.4 kBq or 18.5 kBq of 225Ac-NM600. ** p<0.01, *** p<0.001, ****p<0.0001. * in red denote statistical significance when compared to controls.

**Supplementary Table 3.** Summary of statistical significances of cytokine/chemokine panel between therapeutic groups and controls in TRAMP-C1 tumor bearing animals.


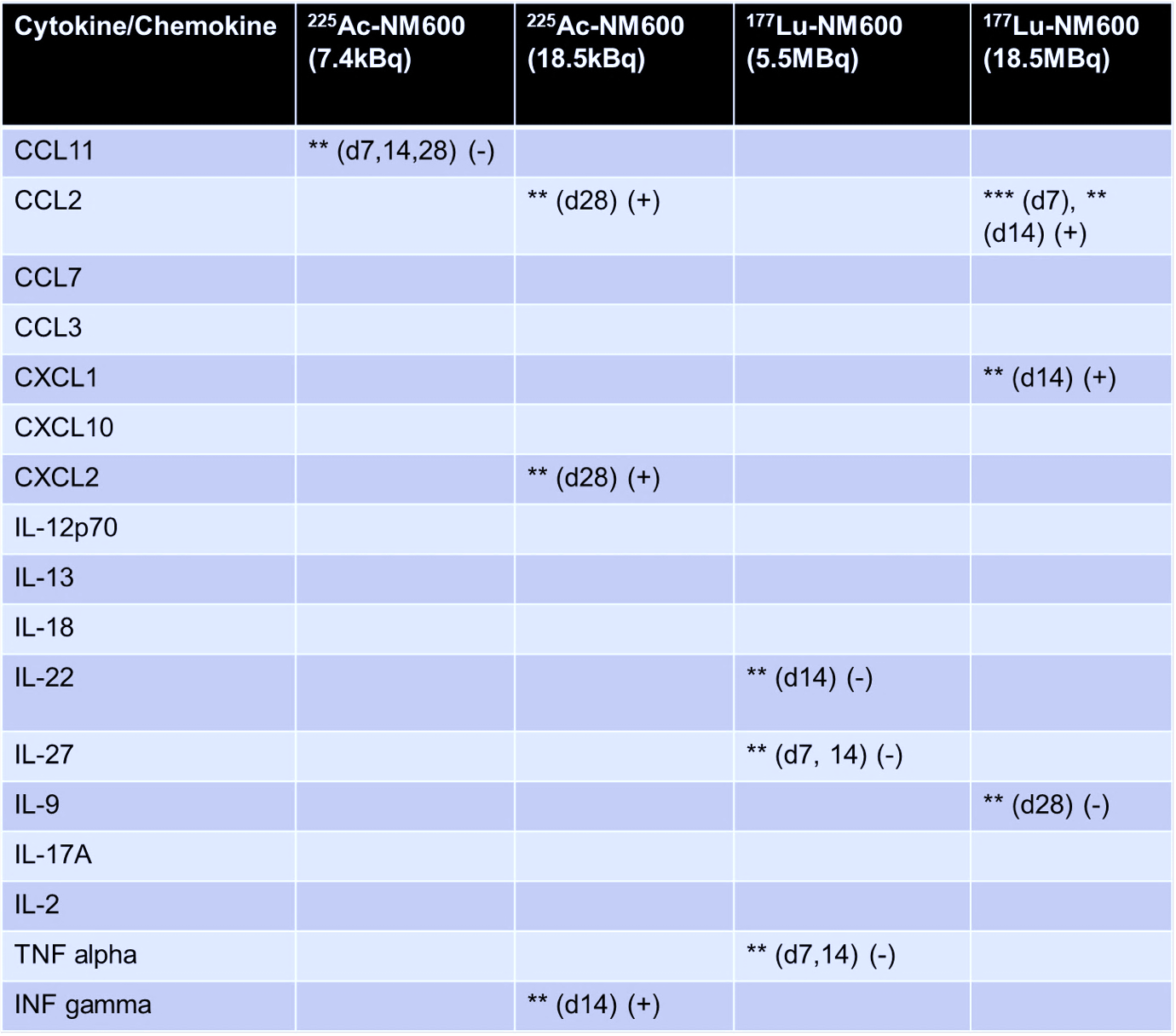


** p<0.01, *** p<0.001. When compared to controls, increased values are denoted with (+) and decreased values with (-).


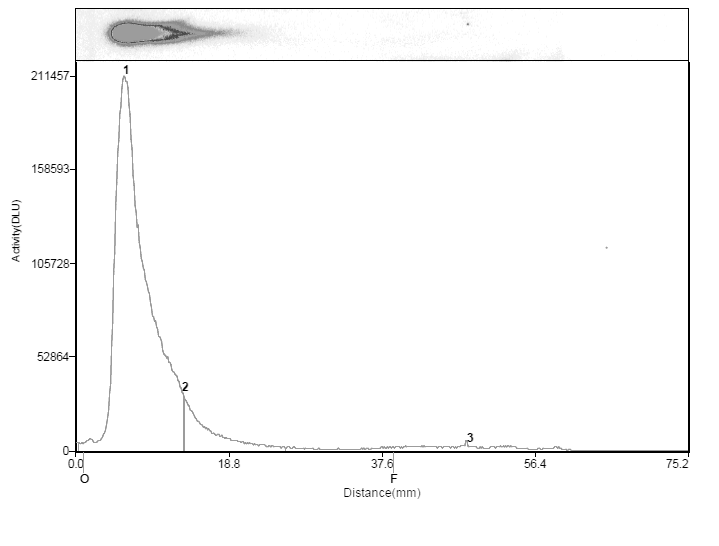


**A)**

**B)**

**Supplementary Figure 7.** Radiolabeling yield and serum stability of ^225^Ac-NM600. **(A)** A representative image of an iTLC and analyzed chromatogram of ^225^Ac-NM600. **(B)** Longitudinal Stability of ^225^Ac-NM60 in human serum.
